# Comparison of a Modified Trabeculectomy Technique With Standard Trabeculectomy in Respect to Effectivity and Complication Rates

**DOI:** 10.1155/joph/2758247

**Published:** 2026-08-18

**Authors:** Suzan Doğruya, Ömer Can Kayıkçıoğlu, Özcan Rasim Kayıkçıoğlu

**Affiliations:** ^1^ Department of Ophthalmology, Uşak University, Uşak, Turkey, usak.edu.tr; ^2^ Department of Ophthalmology, Başkent University, İzmir, Turkey, baskent.edu.tr; ^3^ Ophthalmology, Private Practice, Manisa, Turkey

**Keywords:** glaucoma, intraocular pressure, modified trabeculectomy

## Abstract

**Purpose:**

To compare the results of modified trabeculectomy with a central scleral gutter under the flap and releasable suture with standard trabeculectomy for open angle glaucoma cases.

**Design:**

Retrospective.

**Method:**

This retrospective comparative case series evaluated the outcomes of modified trabeculectomy in 32 eyes and standard trabeculectomy in 44 eyes with primary open‐angle glaucoma or pseudoexfoliative glaucoma. Success was defined as achieving an intraocular pressure (IOP) ≤ 21 mmHg with or without the use of IOP‐lowering eyedrops and no need for additional glaucoma surgery.

**Results:**

There were 20 men (62.5%) and 12 women (37.5%), with a mean age of 61.9 ± 10.5 years in the modified trabeculectomy group and 27 men (61.4%) and 17 women (38.6%), with a mean age of 62.3 ± 9.8 years in the standard trabeculectomy group. Modified trabeculectomy group had a preoperative IOP of 26.9 ± 8.1 mmHg and a postoperative IOP of 15.3 ± 4.1 mmHg (*p* < 0.001), with an average follow‐up period of 38.5 ± 17.2 months. Patients undergoing standard trabeculectomy had a preoperative IOP of 26.7 ± 8.7 mmHg and a postoperative IOP of 14.5 ± 4.6 mmHg (*p* < 0.001), with an average follow‐up period of 42.4 ± 9.4 months. The success rate was 97.7% in the standard trabeculectomy group and 96.8% in the modified trabeculectomy group. In the modified trabeculectomy group, 30 (93.7%) had no complications, compared to 33 (75%) in the standard trabeculectomy group (*p* = 0.03). Anterior chamber shallowing was observed in 2 eyes (6.3%) in the modified trabeculectomy group, whereas the standard trabeculectomy group exhibited a broader complication profile, including anterior chamber shallowing in 4 eyes (9.0%), needling revision in 3 eyes (6.8%), hyphema in 2 eyes (4.5%), and choroidal detachment in 2 eyes (4.5%).

**Conclusion:**

The modified trabeculectomy surgery lowers IOP effectively with a lower incidence of postoperative surgical complications.

## 1. Introduction

Trabeculectomy, which was first described by Cairns in 1968, is still the gold standard for surgical treatment of glaucoma [1–4]. In current surgical technique, antimetabolites such as fluorouracil (5‐FU) and mitomycin C (MMC) regulate wound healing, reduce subconjunctival fibrosis, and keep the bleb active for a long time [5–8]. However, administration of antimetabolites has also been associated with complications such as vision‐threatening hypotonia, early and late bleb leak, and endophthalmitis [9–11]. Although trabeculectomy surgery effectively reduces intraocular pressure (IOP), its serious complication rate and low safety profile have led to the search for alternative surgical procedures [12–18].

In our study, we described a modified trabeculectomy technique and evaluated its surgical outcomes and postoperative complications in comparison with standard trabeculectomy.

Despite the well‐established efficacy of trabeculectomy in lowering IOP, postoperative complications—particularly hypotony‐related events—remain a major limitation, especially in the era of antimetabolite use. Various surgical modifications have been proposed to improve the safety profile of trabeculectomy; however, no consensus has been reached regarding the optimal balance between effective filtration and postoperative safety.

The present study was designed to evaluate whether a modified trabeculectomy technique incorporating a central scleral gutter beneath the scleral flap combined with a releasable suture provides comparable IOP control while reducing postoperative complications compared with standard trabeculectomy. We hypothesized that this modification may improve postoperative safety without compromising surgical efficacy.

## 2. Materials and Methods

This is a retrospective study of 76 patients diagnosed with primary open‐angle glaucoma (POAG) or pseudoexfoliative glaucoma (PEXG) who had medically uncontrolled IOP ranging between 21 and 40 mmHg despite treatment and demonstrated progression of visual field defects and/or optic nerve cupping. The study included patients who underwent surgery between January 2014 and January 2020.

All patients underwent a comprehensive ophthalmic examination preoperatively and at each postoperative visit. Best‐corrected visual acuity was measured using a Snellen chart and subsequently converted to the logarithm of the minimum angle of resolution (logMAR) scale for statistical analysis. In cases in which visual acuity could not be reliably measured using the Snellen chart (e.g., counting fingers or hand motion), established logMAR equivalents were applied.

IOP was measured using Goldmann applanation tonometry by the same experienced examiner under standardized conditions. Slit‐lamp biomicroscopy was performed to evaluate the anterior segment, and dilated fundus examination was conducted using indirect ophthalmoscopy to assess the optic nerve head and posterior segment. Gonioscopy was performed preoperatively to confirm an open anterior chamber angle in all included eyes.

Visual field assessment was performed using standard automated perimetry, and glaucomatous progression was defined based on documented deterioration in visual field parameters and/or progressive optic nerve head cupping on clinical examination. All surgeries were carried out by a single glaucoma surgeon with experience in the field (ORK).

Patients diagnosed with POAG or PEXG were included in the study. The diagnosis of POAG was based on the presence of an open anterior chamber angle on gonioscopy, characteristic glaucomatous optic nerve head changes, corresponding visual field defects, and elevated or progressive IOP despite maximally tolerated medical therapy. PEXG was diagnosed based on the presence of pseudoexfoliative material on the anterior segment structures in combination with open‐angle glaucoma features.

Inclusion criteria comprised medically uncontrolled IOP ranging between 21 and 40 mmHg, documented progression of optic nerve damage and/or visual field loss, and indication for primary trabeculectomy. Patients with angle‐closure glaucoma, neovascular glaucoma, uveitic glaucoma, congenital glaucoma, or secondary glaucomas other than pseudoexfoliation were excluded.

Surgical success was defined as achieving a final IOP of ≤ 21 mmHg with or without the use of topical IOP‐lowering medications and without the need for additional glaucoma surgery during follow‐up. Complete success was defined as achieving an IOP of ≤ 21 mmHg without antiglaucoma medication, whereas qualified success referred to IOP control ≤ 21 mmHg with supplemental medical therapy.

This definition was chosen in accordance with commonly used criteria in major trabeculectomy studies, including the Tube Versus Trabeculectomy (TVT) Study and other large glaucoma surgical series, to allow comparability with previously published outcomes [19, 20].

### 2.1. Surgical Technique

All surgeries were performed under retrobulbar anesthesia. A fornix‐based conjunctival flap was created without vertical relaxing incisions in both groups.

### 2.2. Standard Trabeculectomy Group

In the standard trabeculectomy group, a 3 × 4 mm rectangular, limbus‐based partial‐thickness scleral flap was created at approximately one‐third of the scleral depth. Antimetabolite, MMC (0.2 mg/mL), was applied under the conjunctiva over the scleral flap for 2 min using soaked sponges, and then it was thoroughly irrigated.

The anterior chamber was entered through a standard corneal paracentesis at the temporal limbus, and viscoelastic material was injected to maintain anterior chamber depth. Trabecular tissue was excised using a goniopunch. After performing a peripheral iridectomy, the scleral flap was sutured using four 10‐0 nylon sutures, and the conjunctiva was sutured using 8‐0 Vicryl sutures.

### 2.3. Modified Trabeculectomy Group

**FIGURE 1:**
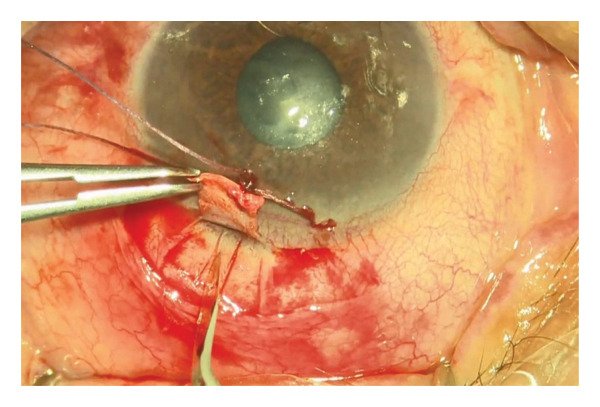
The creation of a limbus‐based rectangular scleral flap 1/3 of the thickness of the sclera.

**FIGURE 2:**
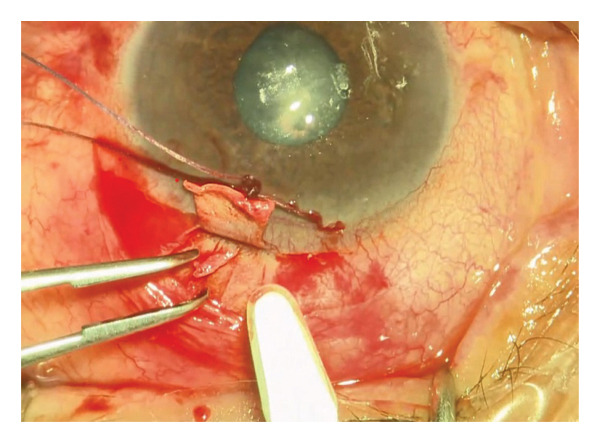
Scleral tissue 1/3 of the thickness of the scleral bed under the scleral flap is removed, and a groove is formed.

In the modified trabeculectomy group, the initial steps were identical to the standard technique: a fornix‐based conjunctival flap and a 3 × 4 mm superficial scleral flap were created, followed by MMC application (Figure 1). The modification involved removing approximately one‐third of the residual scleral bed beneath the initial flap to create an additional 1 × 3 mm gutter (Figure 2). This deeper dissection was designed to modulate aqueous humor outflow dynamics by creating a potential subscleral filtration space beneath the scleral flap, thereby promoting a more controlled and gradual filtration rather than increasing overall outflow.

**FIGURE 3:**
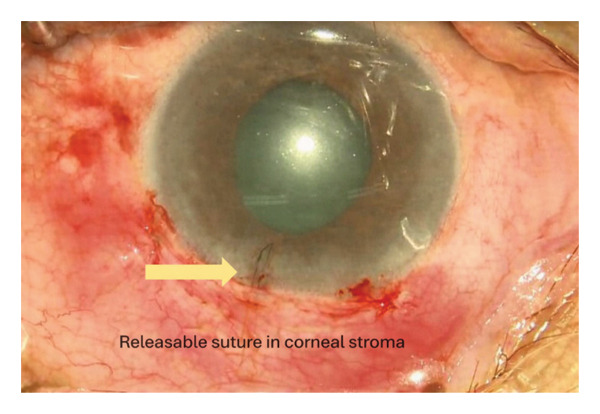
The arrowhead indicates the releasable suture, which is buried in the corneal stroma in the form of a loop.

As in the modified group, the anterior chamber was accessed via a corneal paracentesis, and viscoelastic was injected. Trabecular meshwork tissue was excised with a punch, and a peripheral iridectomy was performed. The scleral flap was secured with four 10‐0 nylon sutures at the sides and one releasable suture in the middle of the rectangular flap loop, which was embedded in the limbal cornea. The conjunctiva was reapproximated/sutured using 8‐0 Vicryl sutures (Figure 3). The viscoelastic agent in the anterior chamber was not removed at the end of the procedure.

In the modified trabeculectomy group, a single releasable suture was used along with four fixed scleral flap sutures. In the standard trabeculectomy group, only fixed sutures were applied, and argon laser suture lysis was performed when needed during the follow‐up. Ocular massage was used often to augment bleb functions. Postoperatively, cyclopentolate 1% was applied three times per day, and a combination of moxifloxacin and dexamethasone was initiated with moxifloxacin administered four times daily and dexamethasone six times daily. This postoperative regimen, including ocular massage when indicated, cyclopentolate, moxifloxacin, and dexamethasone, was applied uniformly to both the standard and modified trabeculectomy groups. The steroid was then tapered gradually and discontinued over a period of 6 weeks. The patients were followed up on the first day, first month, third month, sixth month, 1 year, and longer when possible. If the IOP was high, the releasable suture was removed on the first postoperative days. If not, it was removed after at least 2 weeks of control.

Postoperative IOP was measured at standardized follow‐up visits, including postoperative day 1, 1 month, 3 months, 6 months, 12 months, and the final follow‐up visit. IOP values at 1 month postoperatively and at the end of follow‐up were used for outcome analyses, as reported in the Results section.

Surgical failure was defined as an IOP greater than 21 mmHg on two consecutive follow‐up visits at least 3 months postoperatively, a persistent loss of two or more lines of Snellen visual acuity beyond 1 month after surgery, and/or the occurrence of significant surgical complications requiring reoperation. The use of IOP‐lowering eyedrops postoperatively was not defined as surgical failure, provided that the final IOP remained ≤ 21 mmHg without the need for additional surgical intervention.

Postoperative complications included hyphema, shallow anterior chamber causing corneal endothelial damage, severe hypotony (IOP < 5 mmHg), and choroidal effusion. Mild, transient hyphema resolving within 1 week was not considered surgical failure. Major complications that were considered as surgical failures included endophthalmitis, persistent hypotony, and other vision‐threatening conditions which we did not encounter.

This study was retrospective and nonrandomized. Surgical technique allocation was based on a chronological approach rather than patient‐specific clinical characteristics. Standard trabeculectomy was performed during the earlier period of the study, and following refinement of the surgical technique, all subsequent cases underwent the modified trabeculectomy procedure. No selection criteria based on glaucoma subtype, baseline IOP, disease severity, or other clinical findings were used to preferentially assign patients to either surgical technique.

The study protocol was approved by the Manisa Celal Bayar University Ethics Committee (approval number: 229; approval date: December 13, 2021), and written informed consent was obtained from all adults participating in this study. The Helsinki Declaration principles were followed.

### 2.4. Statistical Analysis

The SPSS 22.0 package program was used for statistical analysis of the study (SPSS, Inc., Chicago, IL, USA). Descriptive statistical data are presented as the mean and standard deviation in data evaluation. Continuous variables were compared between groups using the independent samples *t*‐test or the Mann–Whitney *U* test, as appropriate. Categorical variables, including the presence or absence of postoperative complications, were compared using the chi‐square test. The results were considered statistically significant at *p* < 0.05.

Correlation analyses between IOP and visual acuity (logMAR), as well as other clinical variables, were performed using Spearman’s rank correlation coefficient.

## 3. Results

There were 20 men (62.5%) and 12 women (37.5%) in the group of 32 patients who had modified trabeculectomy, with a mean age of 61.9 ± 10.5 years, 19 right eyes, and 13 left eyes. All patients’ medical histories were unremarkable. There were 20 patients with POAG and 12 with PEX glaucoma. Standard trabeculectomy was performed on 44 patients; 27 men (61.4%) and 17 women (38.6%), with a mean age of 62.3 ± 9.8 years, 27 right eyes, and 17 left eyes. All patients’ medical histories were unremarkable. There were 29 eyes with POAG and 15 eyes with PEX glaucoma. All patients in both groups received MMC intraoperatively. Five patients in the standard trabeculectomy group and seven patients in the modified trabeculectomy group were pseudophakic preoperatively.

Preoperatively, in the modified trabeculectomy group, mean visual acuity was 0.57 ± 0.32 logMAR, and mean c/d ratio was 0.76 ± 0.21. Preoperatively, in the standard trabeculectomy group, mean visual acuity was 0.50 ± 0.35 logMAR, and mean c/d ratio was 0.79 ± 0.12.

Patients who underwent modified trabeculectomy had a mean preoperative IOP of 26.9 ± 8.1 mmHg and a postoperative IOP of 15.3 ± 4.1 mmHg after a mean follow‐up of 38.5 ± 17.2 months (*p* < 0.001). The number of preoperative IOP‐lowering medications decreased from 3.1 ± 1.0 to 0.6 ± 1.2 after surgery (*p* < 0.001). The decrease in IOP and the number of IOP‐lowering medications were found to be statistically significant. The postoperative drug‐free period was 7.9 ± 9.7 months in the modified trabeculectomy group and 5.7 ± 5.1 months in the standard trabeculectomy group. Independent samples *t*‐test analysis revealed no statistically significant difference between the two groups (*p* = 0.25). In 30 eyes (93.7%), there were no complications. Anterior chamber shallowing developed in two eyes, accounting for 6.3% of cases. Laser suture lysis was performed on the corner sutures in three patients in the modified trabeculectomy group to optimize postoperative aqueous outflow when needed.

The mean preoperative IOP of patients who underwent standard trabeculectomy decreased from 26.7 ± 8.7 mmHg to 14.5 ± 4.6 mmHg at the end of a mean follow‐up of 42.4 ± 9.4 months (*p* < 0.001). The number of preoperative IOP‐lowering medications decreased from 3.3 ± 1.0 to 0.8 ± 1.3 postoperatively (*p* < 0.001). Both reductions were statistically significant. Complications occurred in 11 patients (25%) in the standard trabeculectomy group. Shallow anterior chamber requiring intervention was observed in four eyes (9%), needling revision in three eyes (6.8%), hyphema in two eyes (4.5%), and choroidal detachment in two eyes (4.5%). Three needling revisions were required due to encapsulated blebs with uncontrolled high IOP. All needling revision procedures were performed using needling combined with subconjunctival 5‐FU injections. No complications were observed in 75% of eyes in the standard trabeculectomy group, compared with 93.7% of eyes in the modified trabeculectomy group. The modified trabeculectomy group demonstrated a significantly lower complication rate than the standard trabeculectomy group (*p* = 0.03).

At the last examinations of the modified trabeculectomy patients, IOP was ≤ 21 mmHg in 24 eyes (75%) without glaucoma medication. The success rate for achieving a final IOP ≤ 21 mmHg with supplemental medication was 96.8% in the modified trabeculectomy group. The postoperative mean number of IOP‐lowering medications used was 0.62 ± 1.18. None of the patients had a postoperative IOP of less than 6 mmHg hyphema, or lost their sense of light. However, anterior chamber shallowing occurred in two patients in the modified trabeculectomy group.

At the last exam of standard trabeculectomy patients, IOP was ≤ 21 mmHg in 31 eyes (70.5%) without glaucoma medication. The success rate for achieving a final IOP ≤ 21 mmHg with supplemental medication was 97.7% in the standard trabeculectomy group. The postoperative mean number of IOP‐lowering medications used was 0.77 ± 1.29. None of the patients had a postoperative IOP of less than 6 mmHg or lost their perception of light.

No additional IOP‐lowering medications were used to achieve IOP of ≤ 21 mmHg in 70.5% of the standard trabeculectomy group and 75% of the modified trabeculectomy group. There was no statistically significant difference between the two groups (*p* = 0.34).

In our study, no statistically significant differences in age, visual acuity, cup‐disc ratios, the number of IOP‐lowering medications used before and after surgery, the last IOP, and the follow‐up period were found between the two groups (*p* > 0.05).

The clinical characteristics of patients who underwent modified trabeculectomy and standard trabeculectomy are shown in Table 1 (Table 1).

**TABLE 1:** Clinical preoperative characteristics of patients undergoing modified and standard trabeculectomy.

Variable	Modified trabeculectomy (*n* = 32)	Standard trabeculectomy (*n* = 44)	*p* value
Mean Age (years)	61.9 ± 10.5	62.3 ± 9.8	0.633
Gender (F/M)	12 (37.5)/20 (62.5)	17 (38.6)/27 (61.4)	0.854
Glaucoma type (POAG/PEXG)			0.849
POAG	20 (62.5)	29 (65.9)	
PEXG	12 (37.5)	15 (34.1)	
Visual acuity (logMAR)	0.57 ± 0.32	0.50 ± 0.35	0.920
C/D rate	0.76 ± 0.21	0.79 ± 0.12	0.445
Preop IOP (mmHg)	26.9 ± 8.1	26.7 ± 8.7	0.651
Postop IOP (mmHg)	15.3 ± 4.1	14.5 ± 4.6	0.498
Follow‐up (months)	38.5 ± 17.2	42.4 ± 9.4	0.106
Preoperative IOP‐lowering medications (n)	3.1 ± 1.0	3.3 ± 1.0	0.416
Postoperative IOP‐lowering medications (n)	0.6 ± 1.2	0.8 ± 1.3	0.611
Any postoperative complication, *n* (%)	2 (6.3) anterior chamber shallowing	4 (9.0) anterior chamber shallowing 3 (6.8) bleb revision 2 (4.5) hyphema 2 (4.5) choroidal detachment	**0.03**

*Note:* Values are given as number (percent) or mean ± SD. F: female, M: male, C/D: cup‐to‐disc ratio, PEXG: pseudoexfoliative glaucoma. Values are presented as mean ± standard deviation or number (percentage). Continuous variables were compared using the independent samples *t*‐test or Mann–Whitney *U* test, as appropriate. Categorical variables were compared using the chi‐square test. The bold value in Table 1 indicates statistical significance (*p* < 0.05).

Abbreviation: POAG, primary open‐angle glaucoma.

Correlation analyses between IOP, visual acuity (logMAR), and clinical variables were performed in the overall study population. A weak negative correlation was observed between age and postoperative IOP (Spearman’s *ρ* = −0.237, *p* = 0.034). No other significant correlations were identified. The results are summarized in Supporting Table 1.

## 4. Discussion

In the present study, modified trabeculectomy surgery was found to be as effective as standard trabeculectomy in achieving IOP control. Notably, the modified technique was associated with a significantly lower incidence of postoperative complications. Although all patients were operated on in a tertiary referral center under inpatient conditions, it is important to consider that postoperative complications may be more challenging to manage in outpatient glaucoma surgery settings. In current clinical practice, the reported success rates of glaucoma filtration surgeries vary widely, ranging from 71% to 100%, depending on surgical technique, the underlying etiology of glaucoma, and the definitions of surgical success adopted in the literature [7, 19–22]. Although we used MMC in our surgeries, complication rates were acceptable especially in the modified trabeculectomy group which is comforting for both the patient and the surgeon. While overall postoperative complications were recorded, only events that are likely to be influenced by surgical technique—such as anterior chamber shallowing or hypotony—were considered directly related to the surgical modification.

Recent large‐scale and prospective studies provide updated estimates of trabeculectomy success in contemporary clinical practice. In a randomized multicenter trial comparing trabeculectomy with ab‐externo microshunt implantation, the 2‐year surgical success rate (defined as ≥ 20% IOP reduction without increased medications) was significantly higher in the trabeculectomy group (64.4%) compared to microshunt eyes (50.6%) [23]. Wagner et al. reported long‐term outcomes after trabeculectomy in open‐angle glaucoma, demonstrating that qualified surgical success was achieved in approximately 73% of eyes and complete success in around 69% at a mean follow‐up of 6 years, supporting durable efficacy of the procedure [24]. Additionally, retrospective analyses such as Vastardis et al. have shown that trabeculectomy yields superior absolute success rates compared to other filtering procedures, with significant IOP reduction and decreased medication burden at intermediate follow‐up points [25]. These contemporary data emphasize that trabeculectomy continues to offer high and stable success rates in current practice, despite evolving surgical techniques and the increasing use of minimally invasive alternatives.

Several previous studies have shown that trabeculectomy effectively lowers IOP in patients with open‐angle glaucoma. Consistent with these findings, our study demonstrates that both surgical approaches yielded substantial and sustained IOP reductions over long‐term follow‐up. However, the modified technique was associated with a significantly higher rate of complication‐free outcomes. The releasable suture represents the primary mechanism for early postoperative IOP control, allowing for titration of aqueous outflow and reducing the risk of early hypotony. In contrast, the scleral gutter should be regarded as a structural modification that may influence the distribution and stability of aqueous humor outflow rather than acting as a direct hypotony‐preventive mechanism. Consistent with this interpretation, no significant difference in early postoperative IOP was observed between the two groups, underscoring that the observed reduction in complications cannot be attributed solely to an early pressure‐lowering effect of the scleral gutter.

The use of a releasable suture enables individualized postoperative adjustment of IOP, particularly in eyes treated with MMC, where the risk of overfiltration is increased. Timely removal of the suture based on IOP and clinical response allows for a controlled transition from restricted to more permissive aqueous outflow. This controlled approach, in combination with the subscleral space created by the scleral gutter, may contribute to a more stable postoperative filtration environment. However, the relative contribution of each component cannot be determined within the current study design.

The key modification distinguishing the modified trabeculectomy technique from the standard approach lies in the management of the scleral bed beneath the superficial scleral flap. The modified trabeculectomy group underwent a further step in which an additional 3 × 1.5 mm area of scleral tissue removal—corresponding to one‐third of the residual scleral thickness—was excised beneath the superficial flap. This created a more pronounced subscleral filtration space, intended to facilitate more controlled aqueous outflow during the early postoperative period rather than lowering final IOP, thereby potentially contributing to a more stable postoperative healing environment. Although nonpenetrating deep sclerectomy techniques, such as those reported by Xu et al., aim to reduce hypotony‐related complications through controlled filtration, our surgical modification differs fundamentally in that it preserves a penetrating trabeculectomy framework while incorporating a limited subscleral space to enhance postoperative safety [26].

The scleral gutter may function as a subscleral reservoir that modulates aqueous humor outflow dynamics both in the early and late postoperative periods. In the early phase, the presence of this additional space may help distribute aqueous humor more gradually beneath the scleral flap, thereby buffering sudden increases in outflow and reducing the risk of overfiltration and hypotony‐related complications such as shallow anterior chamber or choroidal detachment.

In the longer term, the gutter may promote a more diffuse filtration pattern by allowing aqueous humor to spread over a broader subscleral area rather than being concentrated at a single focal point. This may reduce localized mechanical stress on the scleral flap and conjunctival tissues, potentially influencing wound healing and limiting excessive fibrosis. As a result, the scleral gutter may contribute to a more stable filtration environment while maintaining effective IOP control. This may explain why similar IOP outcomes were observed despite differences in complication rates.

Although these proposed mechanisms remain theoretical and were not directly evaluated in the present study, they provide a plausible explanation for the lower rate of postoperative complications observed in the modified trabeculectomy group. This combined approach may further contribute to controlled postoperative aqueous outflow and improved safety, particularly in eyes treated with MMC.

Kirwan et al. reviewed 428 patients who had trabeculectomy after a 2‐year follow‐up. Antifibrotic agents were used in 93% of the cases, and the rate of achieving a 20% reduction in preoperative IOP and IOP ≤ 18 mmHg without the use of IOP‐lowering drugs was defined as success, reaching 78%. Suture manipulation (43%), re‐suturation for hypotonia (7%), bleb needling (17%), and cataract extraction (31%) were all performed postoperatively. On the Snellen chart, 28% had two‐line vision loss, 7.2% had late‐onset hypotonia, and 14% had bleb leakage. Bleb‐associated endophthalmitis developed 1 month after surgery in one patient and 3 years later in another due to subsequent cataract surgery [27]. In our study, MMC was used in all patients in the modified trabeculectomy group, and IOP ≤ 21 mmHg was achieved in 24 (75%) eyes postoperatively without additional antiglaucoma medication.

Cillino et al. reported a success rate of 35.3% (6 eyes) in nonpenetrating deep sclerectomy and 55.5% (10 eyes) in punch trabeculectomy in achieving drug‐free target IOP ≤ 17 mmHg with a mean follow‐up of 22.5 months. Antimetabolite, Nd: YAG laser goniopuncture, and laser suture lysis were not performed on any of the patients. Hyphema and inflammation were more common in the punch trabeculectomy group. Hypotonia was significantly more common in the punch trabeculectomy group than in the nonpenetrating deep sclerectomy group (with or without phaco) (*p* = 0.02, *p* = 0.04). The Punch trabeculectomy group had significantly more anterior chamber shallowness and choroidal detachment (*p* = 0.04, *p* = 0.04) [28]. Punch trabeculectomy was performed in both groups in our study. Only two patients in the modified trabeculectomy group had a shallow anterior chamber, whereas the trabeculectomy group had hyphema in two eyes, needling revision in three eyes, a shallow anterior chamber in four eyes, and choroidal detachment in two eyes.

Gedge et al. compared tube surgery and trabeculectomy in 212 patients. At 3‐year follow‐up, IOP was 13.0 ± 4.9 in the tube group and 13.3 ± 4.9 in the trabeculectomy group (*p* = 0.78), the number of glaucoma drugs was 1.3 ± 1.3 in the tube group, 1.0 ± 1.5 in the trabeculectomy group (*p* = 0.30), the failure rate was 15.1% in the tube group, 30.7% in the trabeculectomy group (*p* = 0.010), and postoperative complications were 39% in the tube group and 60% in the trabeculectomy group.

Tube surgery with MMC was more successful than trabeculectomy during the first 3 years of the TVT study. Both procedures used similar IOP reduction and additional medical therapy. Postoperative complications were more common following trabeculectomy with MMC [29]. Sun et al. found similar IOP reductions in patients who underwent tube shunting or trabeculectomy after trabeculectomy and/or cataract surgery, but trabeculectomy eyes used less glaucoma medication [30]. The success rate in the TVT study, defined as IOP < 21 mmHg, was reported to be 86.5% [19]. At the end of the follow‐up period for modified trabeculectomy patients, IOP was ≤ 21 mmHg in twenty‐four eyes (75%) without the use of glaucoma medication and 96.8% with full treatment. Our complication rate was 6.2%, and the mean number of IOP‐lowering medications used postoperatively was 0.62 ± 1.18. Our findings were comparable with the literature data.

In our study, there was no statistically significant difference between the two groups in terms of age, visual acuity, cup‐disc ratios, number of IOP‐lowering medications used before and after surgery, last IOP, and follow‐up time. There was no statistical difference between the success rate with the use of supplemental antiglaucoma IOP‐lowering medications and the rate of final IOP reaching ≤ 21 mmHg in either group (*p* > 0.05). However, the rate of postop complications after modified trabeculectomy was found to be statistically significantly lower than in the standard trabeculectomy group (*p* = 0.03). The relatively favorable outcomes observed in the modified trabeculectomy group may, in part, be attributed to the specific surgical technique employed. One of the key features of this technique is the closure of the scleral flap with four permanent and one releasable suture. This approach may have contributed to improved postoperative safety by allowing individualized flap tension adjustment during follow‐up. In addition, the space created by the removal of scleral tissue from the trabeculectomy area and the use of a releasable suture may further enhance the controlled drainage of aqueous humor, allowing for more effective and safer pressure regulation.

Furthermore, the space may serve to buffer fluctuations in IOP, promoting a more stable and controlled filtration pattern, which may indirectly reduce the risk of complications such as choroidal effusion and shallow anterior chamber formation.

Recent studies have continued to highlight that, despite advances in surgical technique and postoperative management, trabeculectomy remains associated with a substantial rate of postoperative complications, particularly in eyes treated with antimetabolites. Contemporary series have reported complication rates ranging from approximately 20% to 40%, including shallow anterior chamber, hypotony, choroidal detachment, and bleb‐related interventions, even when modern safer surgery principles are applied. For example, Strzalkowska et al. emphasized that although trabeculectomy remains highly effective, careful surgical modulation is required to balance filtration efficacy with safety in the modern era. Similarly, Martin and Lübke highlighted the critical role of postoperative wound healing modulation and suture management in minimizing early complications. In this context, the lower complication rate observed in the modified trabeculectomy group in our study appears comparable or favorable relative to these recent reports, while maintaining effective IOP control [8, 17].

Therefore, the lower complication rate observed in the modified trabeculectomy group should be interpreted as an association rather than definitive evidence of causality, and further prospective randomized studies are required to isolate the true effect of the surgical modification.

Additionally, the use of a releasable suture technique in modified trabeculectomy groups may have further contributed to minimizing postoperative complications by allowing controlled postoperative IOP titration and reducing the risk of overfiltration/bleb leakage‐related adverse effects.

This study has several limitations. First, its retrospective and nonrandomized design introduces potential selection, chronological, and learning‐curve bias, as the modified technique was preferentially applied after surgical refinement. Although all procedures were performed by the same experienced surgeon, cumulative surgical experience over time may have influenced postoperative outcomes independently of the surgical modification itself.

A key limitation of the present study is that the modified trabeculectomy technique incorporates two simultaneous surgical modifications—namely, the creation of a scleral gutter and the use of a releasable suture—without separate evaluation of their individual effects. Therefore, it is not possible to determine whether the observed reduction in postoperative complications is attributable predominantly to the scleral gutter, the releasable suture, or their combined effect. Given the well‐established role of releasable sutures in early postoperative IOP modulation, it is likely that this component contributed significantly to the improved safety profile. The scleral gutter may provide an additional structural mechanism influencing aqueous outflow dynamics; however, its independent contribution remains uncertain. Future prospective, controlled studies with separate surgical arms are needed to clarify the relative impact of each modification.

Prospective studies with larger patient cohorts undergoing modified trabeculectomy would provide stronger evidence to the literature. In addition, the high prevalence of PEX syndrome in both groups (37.5% in the modified group and 34% in the standard group) may have contributed to lower surgical success and higher complication rates. PEX is known to increase surgical difficulty and negatively impact long‐term outcomes. Therefore, future prospective studies with subgroup analyses based on PEX status are warranted.

In conclusion, although trabeculectomy surgery reduces IOP, the risk of postoperative complications necessitates the development of safer surgical modifications. Modified trabeculectomy was found to provide IOP reduction comparable to standard trabeculectomy, with a lower complication rate, which is of particular importance in the context of outpatient glaucoma surgery.

**Nomenclature**
IOP: Intraocular pressure
POAG: Primary open‐angle glaucoma
PEX: Pseudoexfoliation
VA: Visual acuity
logMAR: Logarithm of the minimum angle of resolution
MMC: Mitomycin C

## Author Contributions

Özcan Rasim Kayıkçıoğlu, Suzan Doğruya, and Ömer Can Kayıkçıoğlu designed the study; Özcan Rasim Kayıkçıoğlu, Suzan Doğruya, and Ömer Can Kayıkçıoğlu conducted the study; Özcan Rasim Kayıkçıoğlu, Suzan Doğruya, and Ömer Can Kayıkçıoğlu managed the data; Özcan Rasim Kayıkçıoğlu, Suzan Doğruya, and Ömer Can Kayıkçıoğlu interpreted the data; Özcan Rasim Kayıkçıoğlu, Suzan Doğruya, and Ömer Can Kayıkçıoğlu prepared the manuscript.

## Funding

The authors received no financial support for the research, authorship, and/or publication of this article.

## Disclosure

All authors have given final approval for this version to be published.

## Ethics Statement

The study protocol was approved by the Manisa Celal Bayar University Ethics Committee (approval number: 229; approval date: December 13, 2021).

## Consent

Written informed consent for publication was obtained from all participants.

## Conflicts of Interest

The authors declare no conflicts of interest.

## Supporting Information

Additional supporting information can be found online in the Supporting Information section.

## Supporting information


**Supporting Information** Supporting Table 1. Correlation analysis between intraocular pressure, visual acuity (logMAR), and clinical variables.

## Data Availability

The datasets used and/or analyzed during the current study are not publicly available due to patient confidentiality but are available from the corresponding author on reasonable request.
