# Supplementary material for: Comparison of a Modified Trabeculectomy Technique With Standard Trabeculectomy in Respect to Effectivity and Complication Rates
Source: J Ophthalmol. 2026 Aug 18;2026:2758247. doi: 10.1155/joph/2758247 (PMC13485356; doi:10.1155/joph/2758247)
Supplement: Supplementary file 1 [file JOPH-2026-2758247-s001.docx]

## Supplementary Table 1. Correlation analysis between intraocular pressure, visual acuity (logMAR), and clinical variables

| **Variable** | **Postoperative IOP (ρ)** | **p value** | **Visual acuity (logMAR) (ρ)** | **p value** |
| --- | --- | --- | --- | --- |
| **Age** | -0.237 | 0.034 | -0.064 | 0.594 |
| **Preoperative IOP** | 0.031 | 0.787 | 0.033 | 0.782 |
| **Cup–disc ratio** | -0.212 | 0.061 | -0.033 | 0.785 |
| **Follow-up duration (months)** | 0.003 | 0.984 | -0.144 | 0.300 |
| **Preoperative IOP-lowering medications** | -0.045 | 0.691 | -0.049 | 0.684 |
| **Postoperative IOP-lowering medications** | 0.092 | 0.419 | -0.005 | 0.966 |

Footnote: Spearman’s rank correlation coefficient was used. Bold values indicate statistically significant correlations (p < 0.05).
